# Virus-like particles vaccines based on glycoprotein E0 and E2 of bovine viral diarrhea virus induce Humoral responses

**DOI:** 10.3389/fmicb.2022.1047001

**Published:** 2022-10-31

**Authors:** Ningning Yang, Jiangwei Zhang, Mingguo Xu, Jihai Yi, Zhen Wang, Yong Wang, Chuangfu Chen

**Affiliations:** ^1^College of Animal Science and Technology, Shihezi University, Shihezi, China; ^2^Intelligent Breeding of Livestock and Poultry, Tiemenguan Vocational and Technical College, Tiemenguan, China; ^3^Key Laboratory of Control and Prevention of Animal Disease, Xinjiang Production & Construction Corps, Shihezi, China; ^4^Co-Innovation Center for Zoonotic Infectious Diseases in the Western Region, Shihezi, China

**Keywords:** bovine viral diarrhea virus, virus-like particles, E0, E2, vaccine

## Abstract

Bovine viral diarrhea/mucosal disease (BVD/MD) is a viral infectious disease that seriously endangers the health of cattle herds and brings serious economic losses to the global cattle industry. Virus-like particles (VLPs) are empty shell structures without viral nucleic acid, which are similar to natural virus particles in morphology and structure. Because of their strong immunogenicity and biological activity, some of them have been used as vaccines in clinical trials. In this study, we developed a strategy to generate BVDV (E0 + E2, E2 + E2) VLPs using an insect baculovirus expression vector system (BEVS). The VLPs obtained were detected by immunofluorescence assay (IFA), western blotting analyses and transmission electron microscope (TEM), and the results showed that VLPs of high purity were obtained. Mice immunized with VLPs (15  μg) and Freund’s adjuvant (100  μl) elicited higher BVDV-neutralizing antibody in comparison with Freund’s adjuvant control (*p* < 0.0001), and even on day 21 or 35 post-prime immunization, the neutralizing antibody levels of mice immunized with E0 + E2 or E2 + E2 VLPs were significantly higher compared with inactivated vaccine (*p* < 0.05). A subsequent challenge reveals that the viral loads of livers, kidneys, spleens, lungs and small intestines were significantly lower compared with control (*p* < 0.0001), and the viral loads of mice immunized with E0 + E2 or E2 + E2 VLPs in the small intestines were significantly lower compared with inactivated vaccine (*p* < 0.05). Thus, VLPs are a promising candidate vaccine and warrants further clinical evaluation.

## Introduction

Bovine viral diarrhea virus (BVDV) is an important pathogen that belongs to the genus *pestivirus* within the family *Flaviviridae*, which is endemic in many parts of the world and poses a great threat to animal husbandry (Peterhans et al., 2010; He et al., 2020). BVDV mainly infects cattle, but it also can infect other animals, including pigs, sheep, deer, camels, etc. (Leng et al., 2022). The animals infected with BVDV mainly show gastrointestinal diseases, fever, cough, reproductive insufficiencies (e.g., infertility, stillborn fetus, abortion), immunosuppression, Persistent infection (PI), mucosal disease, leukopenia and hemorrhagic syndrome (Wang et al., 2021; Yue et al., 2022). The World Organization for Animal Health (WOAH) classifies BVDV into three disease categories based on the degree of harm to animal health and human public health.

Olafson and fox (Olafson, 1946) first found the virus in sick cattle characterized by peptic ulcer in 1946 and dysentery in New York State and successfully isolated the BVDV strain in 1957 (Lee and Gillespie, 1957). According to the *Flaviviridae* Study Group of the International Committee on Taxonomy of Viruses (ICTV), BVDV is classified into three genotypes: *Pestivirus* A (BVDV-1), *Pestivirus* B (BVDV-2), and *Pestivirus* H (BVDV-3) (Smith et al., 2017). BVDV-1 is further classified into 23 subtypes (1a–1w), BVDV-2 is classified into four subtypes (2a–2d), and BVDV-3 is classified into Brazilian source, Thai source and Italy source (Bauermann et al., 2013; Deng et al., 2020; Wang et al., 2021). However, currently only BVDV-1 is often used in laboratory research and vaccines development. BVDV is divided into two biotypes, cytopathic (cp) and non-cytopathic (ncp) according to whether they induce cytopathic effect (CPE) in cells culture. While most *pestiviruses* are ncp, both ncp and cp virus variants have been reported for three genotypes. The BVDV genome is single-stranded RNA of approximately 12.3–13 kb (Simmonds et al., 2017). Virus contains a single, long open reading frame (ORF) flanked by 5′- and 3′-untranslated regions (5′UTR and 3′UTR), which form specific secondary structures required for genome replication and translation (Simmonds et al., 2017). The ORF is further processed into four structural proteins C (capsid protein, core), E0 (envelope protein RNase secreted, Erns), E1 and E2, and eight nonstructural proteins Npro (N-terminal autoprotease), p7, NS2, NS3, NS4A, NS4B, NS5A, and NS5B by virus-encoded and cellular proteases (Meyers and Thiel, 1996).

Antibodies directed to structural proteins E0 and E2 and nonstructural protein NS3 have been demonstrated in infected bovine (Zoth and Taboga, 2006; Chen et al., 2014). E0 and E2 antibodies displayed virus-neutralizing abilities and conferred protection against viral infection (Chen et al., 2014). E0 protein is the first outer membrane glycoprotein encoded by virus genome, and the disulfide bond formed by 8–9 conserved cysteine residues form the skeleton of this protein (Branza-Nichita et al., 2004). E0 protein is often used as diagnostic antigen because it is highly conserved, and it is also used in the development of genetically engineered vaccines because of their good immunogenicity (Zoth and Taboga, 2006; Ren et al., 2020). E2 protein is a receptor protein that binds to the cell surface receptor CD46, and it is also a major protein that produces virus-neutralizing antibodies (Krey et al., 2006; Zezafoun et al., 2011). E2 glycoprotein was expressed using the insect cell expression system to immunize calves, which can effectively protect against the challenge of homologous strains but not of the heterologous strains (Bolin and Ridpath, 1996). Nobiron et al. (2003) immunized mice with DNA vaccine prepared by E2 gene and found that it can induce strong helper T cell (Th1) immune response. Due to the good immunogenicity of E2 protein, so it has become the preferred target protein for the study of vaccines against BVDV.

Vaccine immunization and killing of persistently infected animals are two important means for BVDV prevention, control and purification. As early as 1961, Coggins (Coggins et al., 1961) reported a live attenuated vaccine of BVDV. Since the first BVDV vaccine was reported, many live attenuated and inactivated vaccines have been reported successively. In general, these vaccines are effective in protecting against homologous strains, but not for animals infected with heterologous strains. Because of the shortcomings of traditional vaccines, this prompted researchers to continue to develop a safe and effective BVDV vaccine. With the development and application of molecular biology technology, it provides a broad prospect for the development and production of new BVDV vaccines. At present, many scholars have developed new BVDV vaccines in various ways, including subunit vaccines, recombinant live vector vaccines, DNA vaccines and VLPs vaccines.

Different expression systems can produce VLPs, including *Escherichia coli* (*E. coli*), yeast, baculovirus/insect cells, mammalian cells and plant, etc. However, there are all advantages and disadvantages to using each expression system to produce VLPs. Considering the requirements of protein folding and post-translational modification (PTM), mammalian cells are more suitable for VLPs production. Although mammalian cells produce fewer proteins, they have more complex and accurate PTM capabilities (Zhu, 2012). BEVS has also become a mature production platform of VLPs vaccines and gene therapy vectors (Felberbaum, 2015; Xu et al., 2022). Approved vaccines derived from baculovirus expression, including human use (Cervarix®, Glybera®, Flublok® and Provenge®) and veterinary use (BAYOVAC CSF E2®, Circumvent® PCV, Porcilis® Pesti, Porcilis® PCV and Ingelvac CircoFLEX®) (Felberbaum, 2015; Xu et al., 2022). BEVS platform has many advantages, including manufacturing speed, flexible product design, inherent security and expansibility, which have attracted strong interest of researchers.

The VLPs based on BEVS have been successfully generated for many members of *Flaviviridae*, including hepatitis C virus (HCV; Elmowalid et al., 2007), dengue fever virus (DENV; Suphatrakul et al., 2015), Zika virus (ZV; Dai et al., 2018), West Nile virus (WNV; Rebollo et al., 2018), Japanese encephalitis virus (JEV; Chang et al., 2020). As a member of *Flaviviridae*, VLPs of BVDV have also been reported by Bolin and Ridpath (1996) and Wang et al. (2021). Since both of these VLPs vaccines are constructed against *Pestivirus* A, which can effectively protect against the challenge by homologous but not heterologous strains. Therefore, in this study, we have developed a more effective multivalent vaccine, which can protect against the infection of *Pestivirus* A and *Pestivirus* B. The genes of *Pestivirus* A (BVDV-LC) E0, *Pestivirus* A (BVDV-LC) E2 and *Pestivirus* B (XJ-04) E2 were optimized, and fusion VLPs based on optimized E0 + E2 and E2 + E2 of BVDV genes were constructed by BEVS, and the immunogenicity and protective effect of VLPs vaccines were verified.

## Materials and methods

### Cells and virus culture

Madin–Darby Bovine Kidney (MDBK) and Sf9 cells were purchased from National Collection of Authenticated (Shanghai, China). MDBK cells were cultured in the Dulbecco’s modified Eagle’s medium (DMEM; Gibco, United States) supplemented with 10% fetal bovine serum (FBS; Gibco, United States) at 37°C with 5% CO_2_. Sf9 cells were cultured in the Grace’s Insect Cell Culture Medium (Thermo Fisher Scientific, United States) supplemented with 10% FBS at 28°C without CO_2_. BVDV NADL strain was purchased from China Institute of Veterinary Drugs Control. The virus was amplified in MDBK cells for 72 h, and titration of the 50% tissue culture infective dose (TCID_50_) was performed using the Reed method (Reed and Muench, 1938).

### Animals

All the experimental procedures involving animals were approved by the Animal Experimental Ethical Committee Form of the First Affiliated Hospital of Medical College, Shihezi University. Female BABL/C mice, 6–8 weeks old with mean BW of 20 ± 5 g were purchased from the Animal Experiment Center of Xinjiang Medical University. All of mice were given enough food and water, a 12 h light–dark cycle and 15–20°C, 50% relative humidity.

### Optimization and synthesis of the genes

The sequences of the *Pestivirus* A BVDV-LC E0 protein (GenBank accession number: QCQ84262.1; residues: 271–497) and *Pestivirus* A BVDV-LC E2 protein (GenBank accession number: QCQ84262.1; residues: 693–1,066; E0 + E2), or *Pestivirus* A BVDV-LC E2 protein (GenBank accession number: QCQ84262.1; residues: 693–1,066) and *Pestivirus* B XJ-04 E2 protein (GenBank accession number: ACQ83621.1; residues: 693–1,064; E2 + E2) were optimized according to the codon usage preference of insect cells. The His-tagged protein sequence was added to the front of the E0 + E2 and E2 + E2 sequences respectively, the transmembrane region was removed, the Kozak sequence GCCGCCACC was added to the N-terminus, a stop codon was added to the C-terminus, and the restriction sites of *BamH* I and *EcoR* I (TaKaRa, China) were added. The genes were synthesized and by Gene Universal (Anhui, China), and linked to PMD19-T vector (TaKaRa, China).

### Construction of recombinant Baculovirus

PMD19-T-E0 + E2 and PMD19-T-E2 + E2 were digested by *BamH* I and *EcoR* I, and then ligated to the pFastBac1 vector (Invitrogen, United States) digested by the same enzyme. The correct clones were verified *via* Sanger sequencing and were, respectively, named pFastBac-E0 + E2 and pFastBac-E2 + E2, which were transformed into DH10Bac™ *E. coli* cells (Invitrogen, United States), and followed by the antibiotic selection. After Sanger sequencing and PCR verification, the correct clones were named recombinant rBacmids-E0 + E2 and rBacmids-E2 + E2, respectively. 1 × 10^6^ Sf9 cells were transfected with 1 μg rBacmids-E0 + E2 or rBacmids-E2 + E2 plasmid using Cellfectin II (Invitrogen, United States) according to the manufacturer’s guidelines. After 5–7 days, cells and supernatants were collected, respectively, after obvious CPE appeared, and stored at –80°C.

### Immunofluorescence assay

Sf9 cells were infected with P2 (passage #2) recombinant baculovirus of Bacmids-E0 + E2 or rBacmids-E2 + E2 at a multiplicity of infection (MOI) of 0.5 per well. At 12 h post-infection, the cells were fixed with 4% paraformaldehyde at room temperature (RT) for 10 min. After blocking 5% BSA at 37°C for 2 h, the cells were incubated with fluorescein isothiocyanate (FITC)-conjugated polyclonal anti-BVDV (VMRD, United States) antibody 4°C overnight. Finally, cells were observed under fluorescent microscopy (Zeiss Axioskop-40, Germany).

### Western blotting analyses

Harvested cells and supernatants were separated by 12% SDS-PAGE gels and transferred onto the 0.45 um PVDF membranes (Millipore, United States). After the PVDF membranes had been blocked with TBST containing 5% non-fat dry milk at RT for 2 h and incubated with the His-tag antibody (Solarbio, China; dilution, 1: 2,000) at 4°C overnight. The next day, the HRP-conjugated goat anti-mouse IgG (Solarbio, China; dilution, 1: 5,000) was added to incubate for 2 h after washing with TBST. At last, Signals were detected using a chemiluminescent ECL reagent (Thermo Fisher Scientific, United States).

### Production and purification of VLPs

Sf9 cells were cultured in T225 cell culture flasks, and P2 rBacmids-E0 + E2 or rBacmids-E2 + E2 were inoculated when the cells reached the appropriate density. The obtained cell-containing virus liquid was repeatedly frozen and thawed at −80°C for three times, and then centrifuged at 12,000 rpm, 4°C for 30 min to collect the virus supernatants liquid. For VLPs purification (Chen et al., 2022), the virus supernatants were loaded onto gradient sucrose solution (60, 45, 30, 20, and 10%, from densest to lightest using 6 ml per layer) in 38.5 ml Beckman ultra-clear tubes (Beckman Coulter, United States) and centrifuged at 40,000 rpm, 4°C for 4 h. The target protein in 10–30% was collected, and transferred to the new Beckman ultra-clear tubes containing 10 ml PBS and centrifugated at 40,000 rpm, 4°C for 4 h. The VLPs pellets were resuspended in PBS, and the purity of VLPs proteins were analyzed using Western blot.

### Transmission electron microscope

The concentrated and purified VLPs (20 μl) were dropped onto carbon-coated Cu grids (Beijing Zhongjingkeyi Technology Co., Ltd., China) and incubated at RT for 2 min. The VLPs solution that were not adsorbed were sucked dry with filter paper. The negative stained with 3% phosphotungstic acid was incubated at RT for 5 min. The excess phosphotungstic acid was sucked dry with filter paper and irradiated under infrared lamp for 30 min. TEM HT7700 was used for observation (Hitachi High-Technologies Corporation, Japan).

### Vaccination and challenge of mice.

BALB/c mice (n = 8 per group) were vaccinated *via* subcutaneous (s.c.) injections on days 0 and 14 (Table 1). E2 VLPs were preserved by the zoonotic laboratory of Shihezi University. Sera were collected for neutralization assay at different time points. To assess if vaccination with VLPs can confer protection against BVDV infection, on 42 days after vaccination, we challenged the vaccinated mice with an intraperitoneal inoculation of BVDV NADL at 1.68 × 10^5^ TCID_50_. The viral loads of the livers, spleens, lungs, kidneys and small intestines were detected by qRT-PCR.

**Table 1:** Information on immunization groups.

Groups(*n* = 8)	Dose (μg)	Adjuvant (μl)	Immunization time
Freund’s adjuvant (Control)	15	100	0 d,14^th^ d
E2 VLPs + Freund’s adjuvant	15	100	0 d,14^th^ d
E0 + E2 VLPs + Freund’s adjuvant	15	100	0 d,14^th^ d
E2 + E2 VLPs + Freund’s adjuvant	15	100	0 d,14^th^ d
Commercial vaccine	15	100	0 d,14^th^ d

### Determination of neutralizing antibody titer

Virus neutralization test (VNT) is the gold standard for serological diagnosis of BVDV and classical swine fever virus (CSFV) infection, and it is also used as a reference efficacy test for commercial vaccines (Chimeno et al., 2007; Yi et al., 2022). VNT as previously reported (Hoppe et al., 2019; Wang et al., 2021), the serum samples were heat-inactivated at 56°C for 30 min in a water bath and serially diluted twofold (2^−1^–2^−8^) in DMEM medium, and then an equal volume of DMEM-diluted BVDV virus containing 100 TCID_50_ was added and samples were incubated at 37°C for 2 h. Then the mixtures were removed and cultured with 1% FBS for 5–7 days (37°C, 5% CO_2_), and the cytopathic effects were observed. The 50% neutralizing antibody titer of serum was calculated by Reed method (Reed and Muench, 1938).

### BVDV qRT-PCR

Five of eight vaccinated mice in every group were inoculated intraperitoneal with a mouse-adapted BVDV NADL virus at a dose of 1.68 × 10^5^ TCID_50_ per mouse on day 42 post immunization (Tian et al., 2020). On 15 days after challenge, the liver, kidney, spleen, lung and small intestine from each mouse was removed, and examined for BVDV viral loads. Briefly, the viral RNA was extracted by biospin virus RNA extraction Kit (Bioer Technology, China) and reverse transcribed to synthesize cDNA (CWBIO, China) according to the manufacturer’s guidelines. BVDV 5′UTR gene-specific primers (forward, 5′-CCTAGCCATGCCCTTAGTAGGACT-3′; reverse, 5′-GGAACTCCATGTGCCATGTACA-3′). RT-PCR and melting curve analysis were performed on a Thermofisher ABI 7500 real-time system (Thermo Fisher Scientific, United States). A volume of 10 μl PCR reaction contains 5 μl 2 × Trans Start® Tip Green qPCR Mix (TIANGEN, China), 0.4 μl forward and reverse primers mix (1: 1), 1 μl cDNA template, and 3.6 μl RNase-free water. The reaction conditions involved a 94°C incubation for 5 min, followed by 35 cycles of denaturation at 94°C for 30 s, annealing at 55°C for 30 s, extension at 72°C for 30 s, and final extension at 72°C for 10 min after the last cycle. Viral loads were calculated according to the plot previously standard curve (Tian et al., 2020).

### Statistical analysis

Statistical analyses were performed using GraphPad Prism 8 software (Graph-Pad Software Inc., United States), one-way analysis of variance (ANOVA) or two-way ANOVA was used to determine the differences. All performed experiments were repeated at least three times. *p-values <0.05 were considered statistically significant.

## Results

### Expression of E0 + E2 and E2 + E2 proteins in Sf9 cells

**Figure 1:**
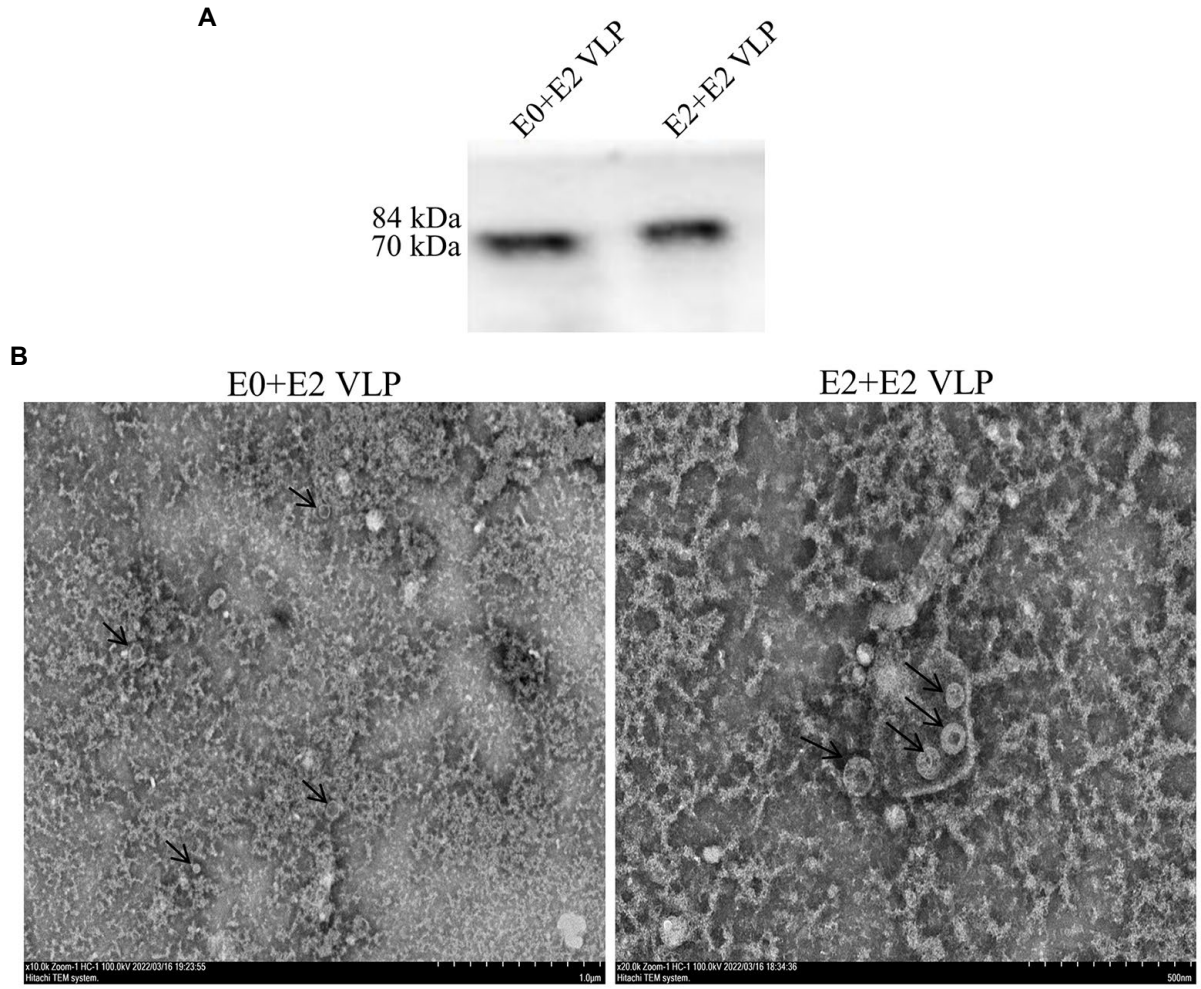
Expression of E0 + E2 and E2 + E2 proteins in Sf9 cells. **(A)** Sf9 cells infected with or without rescued recombinant viruses (rBacmids-E0 + E2 or rBacmids-E2 + E2). NC, Normal Sf9 cells. **(B,C)** Expression of exogenous genes by recombinant baculoviruses identified by IFA and western blotting **(C)**. His-tag antibody (dilution, 1:2,000) as the primary antibody, and HRP-conjugated goat anti-mouse IgG (dilution, 1:5,000) as the secondary antibody.

The E0 + E2 and E2 + E2 genes were optimized, synthesized, and certificated by double enzyme digestion (Supplementary Figure 1). The E0 + E2 and E2 + E2 were inserted into pFastBac1 vector to generate the recombinant plasmid pFastBac-E0 + E2 and pFastBac-E2 + E2 and the subsequent recombinant bacmids (Supplementary Figure 2). The recombinant bacmids were transfected into Sf9 cells and to obtain P1 (passage #1) generation recombinant baculoviruses (Figure 1A). IFA was used to detect the expression of exogenous genes E0 + E2 and E2 + E2 in Sf9 cells. The green fluorescence was observed in under fluorescent inverted microscope (Figure 1B). Expressions of E0 + E2 and E2 + E2 proteins were examined by western blotting analyses. As shown in Figure 1C, expression of E0 + E2 and E2 + E2 proteins were detected in Sf9 cells and supernatants. The above results show that we have successfully obtained recombinant baculoviruses (E0 + E2 and E2 + E2).

### VLPs morphology analysis *via* TEM

**Figure 2:**
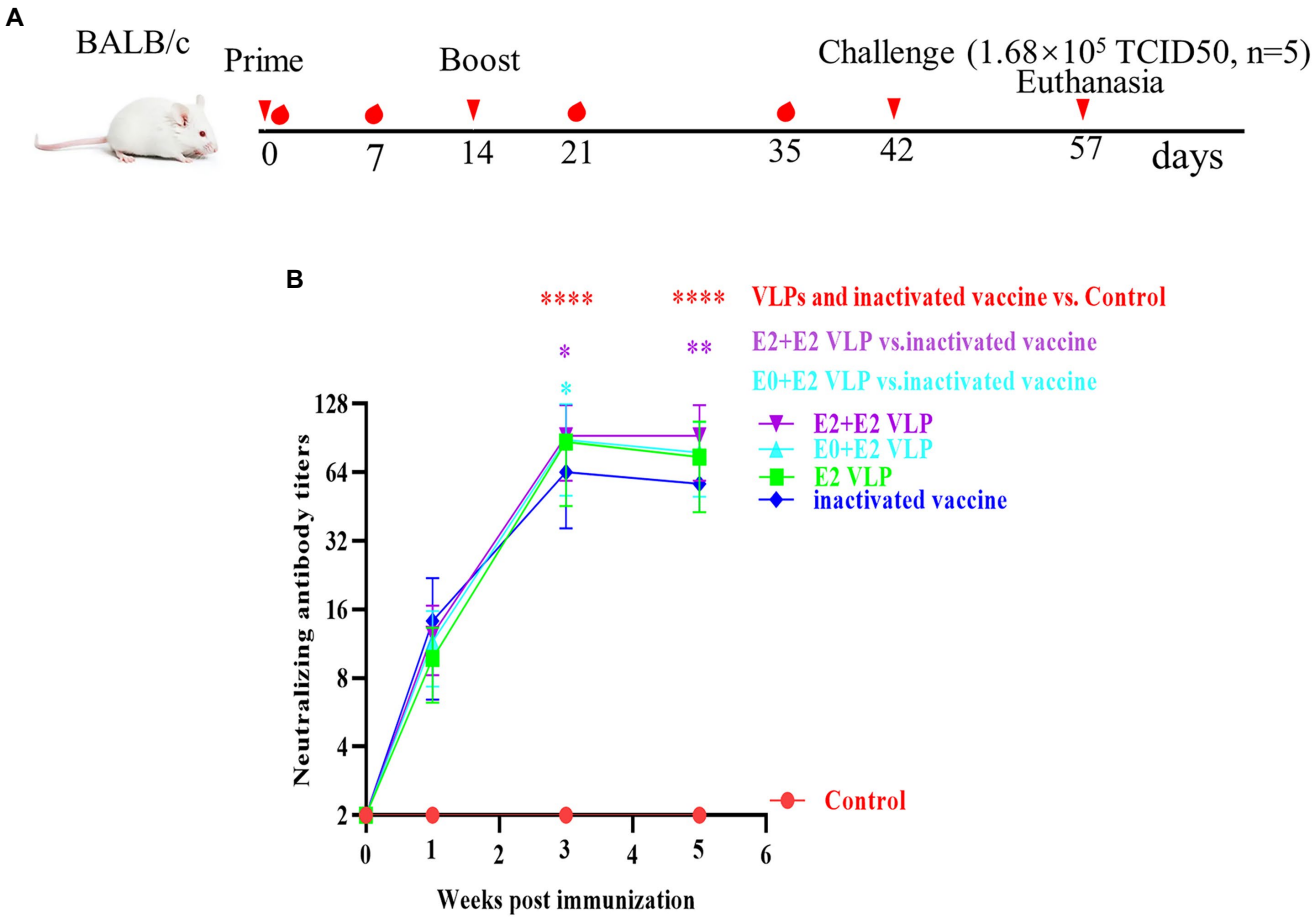
Purification and structure of BVDV-VLPs. **(A)** Sucrose purified VLPs were examined by western blotting. His-tag antibody (dilution, 1:2,000) as the primary antibody, and HRP-conjugated goat anti-mouse IgG (dilution, 1:5,000) as the secondary antibody. **(B)** TEM of negatively stained BVDV VLPs. HT7700 was used for observation of VLPs. VLPs were spherical in morphology, with diameters of about 50–100  nm.

In order to obtain VLPs with high purity, sf9 cells were infected with recombinant baculoviruses, and the proteins were purified by sucrose density gradient ultracentrifugation, followed by western blotting (Figure 2A). TEM HT7700 was used for observation of VLPs. As shown in Figure 2B, the VLPs were spherical in morphology, with diameters of about 50–100 nm, which are similar to the characteristic structure of BVDV virus particles. These results showed that BVDV VLPs are successfully assembled.

### VLPs induce strong neutralizing antibody against BVDV

**Figure 3:**
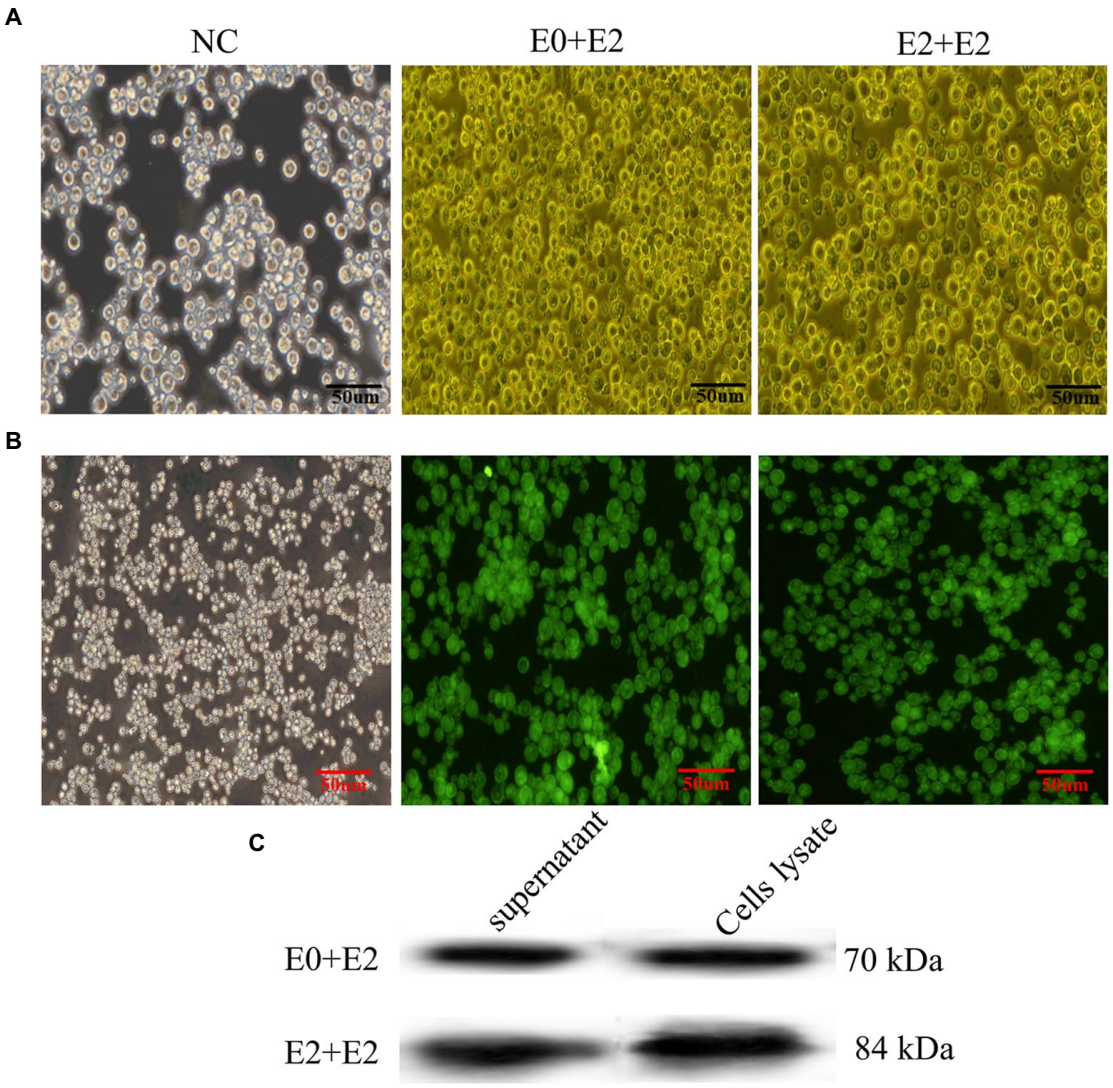
Evaluation of the immunogenicity of the VLP. Mice were primed with Freund’s adjuvant, BVDV VLPs +Freund’s adjuvant or a commercial vaccine and boosted 2 weeks after the prime immunization. Sera were collected of mice at indicated times after the initial vaccination and used to analyze the levels of neutralizing antibody. **(A)** Schematic diagram of immunization and challenge in mice. **(B)** Neutralizing antibody levels in different immunization groups. All performed experiments were repeated at least three times. *p < 0.05, **p < 0.01, ***p < 0.001, and ****p < 0.0001, by two-way ANOVA. Note: red *, VLPs and inactivated vaccine groups vs. control group; purple *, E2+E2 VLP vs. inactivated vaccine and E0+E2 VLP vs. inactivated vaccine.

To determine the neutralizing antibody of BVDV-VLPs, VLPs were used to immunize BALB/c mice, a commercial BVDV inactivated vaccine and Freund’s adjuvant were included as positive and negative controls, respectively (Table 1; Figure 3A). Sera were collected on day 0, 7, 21 and 35 post-prime immunization and BVDV neutralizing antibody were examined by VNT. As shown in Figure 3B, on day 21 and 35 post-prime immunization, the levels of neutralizing antibody in the immunized mice were significantly higher compared with control group (*p* < 0.0001). Importantly, on day 21 or 35 post-prime immunization, the levels of neutralizing antibody in the E0 + E2 or E2 + E2 VLPs immunized mice were significantly higher compared with inactivated vaccine (*p* < 0.05). These results showed that VLPs were able to generate cross-reactivity against BVDV NADL.

### VLPs protect mice against BVDV infection

**Figure 4:**
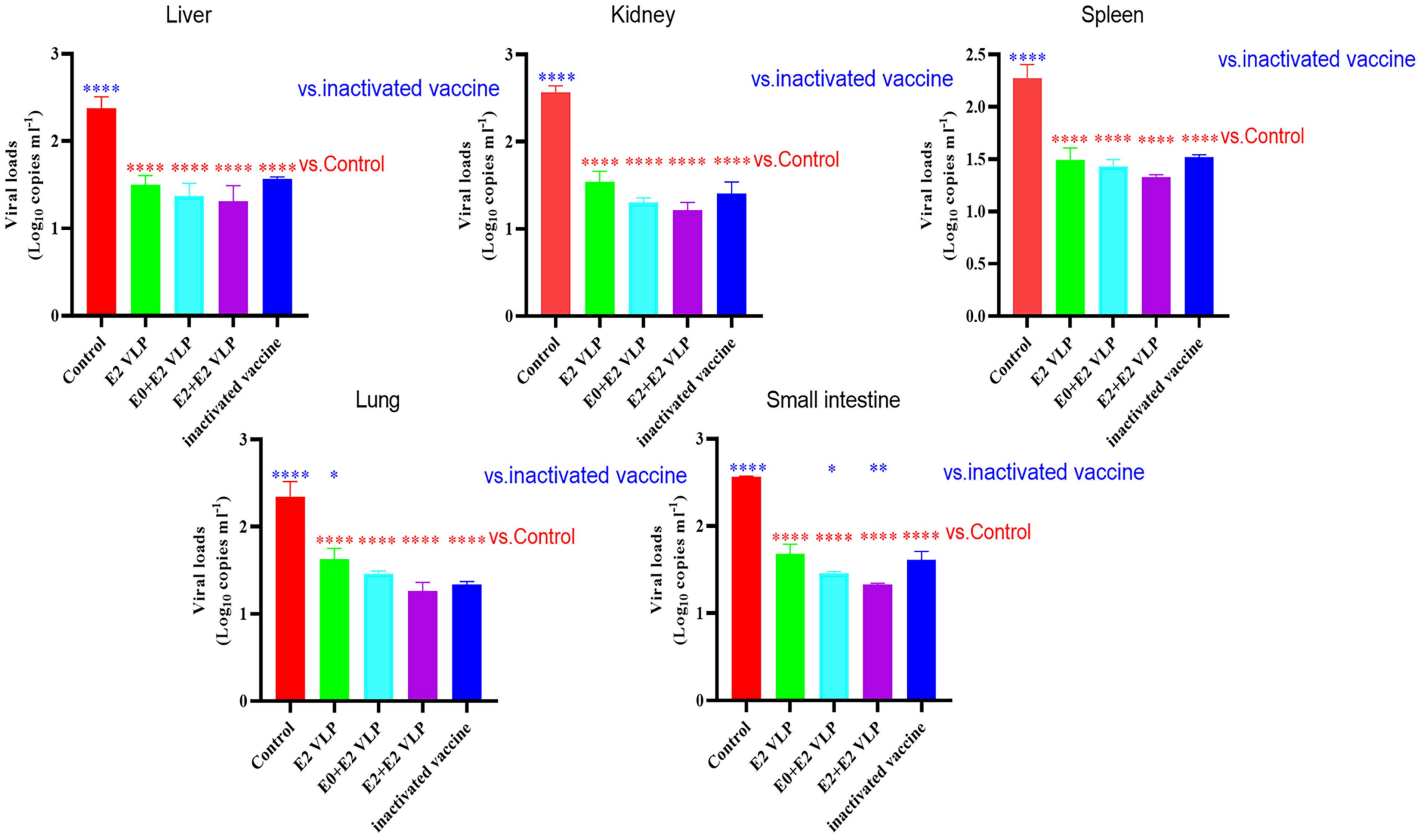
Virus loads detected by qRT-PCR. All performed experiments were repeated at least three times. *p < 0.05, **p < 0.01, ***p < 0.001, and ****p < 0.0001, by one-way ANOVA. Note: blue * VLPs and control groups vs. inactivated vaccine; red * VLPs and inactivated vaccine groups vs. Control group.

qRT-PCR was used to detected the viral loads of livers, kidneys, spleens, lungs and small intestines at 15 days after challenge. The results showed that the viral loads in each tissue were significantly lower compared with control group (Figure 4; *p* < 0.0001). Importantly, the viral loads of mice immunized with E0 + E2 or E2 + E2 VLPs in the small intestines were significantly lower compared with inactivated vaccine (*p* < 0.05). These datas demonstrated that VLPs can better protect mice from NADL strain.

## Discussion

BVDV, CSFV and border disease virus (BDV) belong to the family *Flaviviridae* (Simmonds et al., 2017). Many countries have reported the infection caused by BVDV, so BVDV has caused serious social and economic impacts on the global cattle industry (Richter et al., 2017; Pinior et al., 2019). It is these reasons that some European countries have already launched BVDV eradication programs (Yue et al., 2022). To sum up, vaccination is still an effective measure to prevent BVDV infection. To date, some countries have used traditional live attenuated, inactivated or some combination vaccines to prevent BVDV infection. These traditional vaccines have some disadvantages, such as premature birth, abnormal fetus, insufficient protection, risk of virulence recovery, generation of PI animals, and inability to distinguish vaccine immunity from natural infection. Some BVDV proteins (E0, E2 and NS3) can induce the body to produce detectable antibodies. Among them, E0 and E2 antibodies displayed virus-neutralizing abilities (Zoth and Taboga, 2006). Therefore, the research on BVDV subunit vaccines mainly focuses on E0 and E2. By selecting these two proteins with the ability to neutralize the virus, we may avoid the antibody-dependent enhancement (ADE) produced by the live attenuated vaccines in vaccine development. ADE has been reported in WNV, DENV, Ebola virus (EBOV) and coronavirus infections (Negro, 2020). Although there is no clear evidence that ADE plays a role in the pathogenesis of BVDV, it is vital to avoid ADE in vaccines development.

At present, many scholars have developed novel BVDV vaccines based on E0 and E2 proteins in many ways. These vaccines are a very important tool for implementing BVDV prevention and control, because their use may distinguish vaccine immunity from natural infection, because natural infections can lead to the production of other antibodies, such as NS3 (Zoth and Taboga, 2006). Of course, these vaccines also have some disadvantages, including no or only limited protection efficiency against heterologous strain. However, Chimeno et al. (2007) reported that BEVS was used to express E2 protein of BVDV NADL strain and immunize cattle. The result showed that although complete protection was not observed in cattle, it could cause neutralizing antibody reaction of homologous and heterologous strains *in vitro*. BEVS is far from new. In the past 30 years, researchers have been using this platform to display recombinant proteins, which have been successfully expressed and purified. However, now BEVS has been upgraded from a research tool to a mature manufacturing platform for the production of new biological products (Felberbaum, 2015). In this study, we construct VLPs vaccines based on *Pestivirus* A proteins (E0 and E2) and *Pestivirus* B protein (E2) of BVDV based on BEVS platform. In order to protect *Pestivirus* A and *Pestivirus* B virus infection effectively, and to promote the use in multiple regions in the future.

VaxiJen v2.0 tool was used to analyze the antigenicity of the three proteins (BVDV-LC E0, BVDV-LC E2 and XJ-04 E2). The results showed that the antigenicity of overall prediction for the protective antigen were 0.5302, 0.6059 and 0.4942, respectively, which were higher than that of Threshold for this model: 0.4. ExPASy-ProtParam tool analysis showed that the isoelectric point of the three proteins were 8.12, 8.22 and 6.49, and the instability index (II) were 30.05, 32.67 and 37.96, respectively, which indicated that the three proteins were stable. SignalP 4.1 server and TMHMM Serverv.2.0 software analysis showed that BVDV-LC E0 had no signal peptide and transmembrane domain, and BVDV-LC E2 and XJ-04 E2 proteins had no signal peptide, but there were transmembrane regions at 340–362 and 343–365 amino acids, respectively. Some studies have also found that optimizing codon usage preference can enhance the expression and formation of VLPs (Wang et al., 2020; Yi et al., 2022). Therefore, the E0 + E2 and E2 + E2 fusion genes were synthesized according to the codon usage preference of insect cells and linked to pMD19-T vector, and then linked to pFastBac1 vector after double enzyme digestion. Finally, vaccines based on codon optimized VLPs were obtained.

IFA verified the exogenous genes of E0 + E2 and E2 + E2 could be expressed in Sf9 cells (Figure 1B). In general, VLPs are secretory and need to be purified from cell supernatants rather than cell body lysates. However, our result of western blotting analyses showed that VLPs could be detected in supernatants and cells (Figure 1C). We purified the obtained VLPs by sucrose density gradient centrifugation, and western blotting showed that proteins with high purity (Figure 2A). Finally, TEM was used to verify morphological characteristics of VLPs. These results showed that the size and morphology of VLPs are similar to BVDV (Figure 2B).

Previously, most of the vaccines were reported lacked protection against heterologous strains, while Chimeno et al. (2007) reported that E2 protein expressed by BEVS and immunize cattle can cause of homologous strains and heterologous strains *in vitro* neutralization antibody response. Therefore, we next evaluated the immunogenicity of VLPs in BALB/c mice and their neutralization potential against BVDV NADL strain. VNT is the gold standard for serological diagnosis of BVDV infection, and it is also used as a reference efficacy test for commercial vaccines (Chimeno et al., 2007; Yi et al., 2022). The results of VNT showed that the neutralizing antibody levels of mice immunized with vaccines were significantly higher compared with control (*p* < 0.0001; 3 and 5 weeks), and even the neutralizing antibody levels of mice immunized with VLPs E0 + E2 or E2 + E2 were significantly higher compared with commercial vaccine (*p* < 0.01; 3 or 5 weeks; Figure 3B). The results of VNT showed that VLPs E0 + E2 and E2 + E2 induced higher levels of neutralizing antibody *in vitro*. We further evaluated the protection of mice from challenge, and found that the mice did not die, but the viral loads in the livers, kidneys, spleens, small intestines and lungs of the mice were significantly reduced after immunization (*p* < 0.0001), and the viral loads in the small intestines of the VLPs E0 + E2 and E2 + E2 immunized mice were significantly lower compared with commercial vaccine (*p* < 0.01; Figure 4). In summary, the above results show that VLPs E0 + E2 and E2 + E2 have potential as vaccines, which will increase our confidence in the overall evaluation of the immune effect of VLPs vaccine on cattle.

## Data availability statement

The original contributions presented in the study are included in the article/Supplementary material, further inquiries can be directed to the corresponding author.

## Ethics statement

The animal study was reviewed and approved by All the experimental procedures involving animals were approved by the Animal Experimental Ethical Committee Form of the First Affiliated Hospital of Medical College, Shihezi University

## Author contributions

NY, JZ, MX, and CC conceived and designed the project. NY, JZ, JY, ZW, and YW performed the experiments. NY, JZ, and MX analyzed and interpreted the data and drafted the initial manuscript. CC reviewed and critically revised the initial draft. All authors contributed to the article and approved the submitted version.

## Funding

This work was supported by grants from the Collaborative Innovation Center for the prevention and treatment of high incidence zoonotic infectious diseases in the Western Region (grant no. 2013-179) and the Transformation and Application Demonstration of Rapid Screening Technology Achievements for Important Animal Diseases in Intensive Breeding (grant no. 21322912D).

## Conflict of interest

The authors declare that the research was conducted without any commercial or financial relationships construed as a potential conflict of interest.

## Publisher’s note

All claims expressed in this article are solely those of the authors and do not necessarily represent those of their affiliated organizations, or those of the publisher, the editors and the reviewers. Any product that may be evaluated in this article, or claim that may be made by its manufacturer, is not guaranteed or endorsed by the publisher.
