# Supplementary material for: Virus-like particles vaccines based on glycoprotein E0 and E2 of bovine viral diarrhea virus induce Humoral responses
Source: Front Microbiol. 2022 Oct 31;13:1047001. doi: 10.3389/fmicb.2022.1047001 (PMC9687372; doi:10.3389/fmicb.2022.1047001)
Supplement: Supplementary file 1 [file Table_1.DOCX]

Supplementary Material

# Supplementary Figures and Tables

## Supplementary Figures


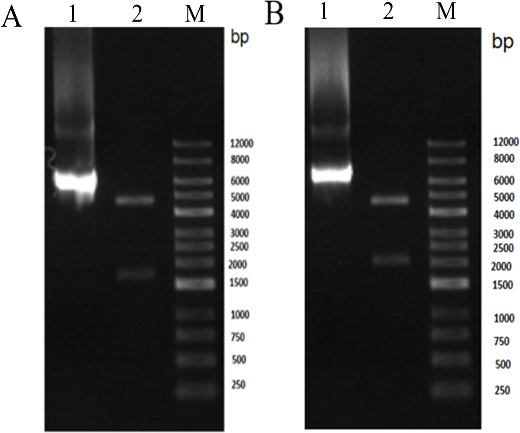


**Supplementary Figure1.** **Double enzyme identification of Recombinant plasmids.** (A) Recombinant plasmid PMD19-T-E0+E2 PCR identification. Lane M：1 kb DNA Marker; Lane 1: Double enzyme digestion of PMD19-T (*BamH* I and *EcoR* I); Lane 2 Double enzyme digestion of PMD19-T-E0+E2 (*BamH* I and *EcoR* I). (B) Recombinant plasmid PMD19-T-E2+E2 PCR identification. Lane M：1 kb DNA Marker; Lane 1: Double enzyme digestion of PMD19-T (*BamH* I and *EcoR* I); Lane 2 Double enzyme digestion of PMD19-T-E2+E2 (*BamH* I and *EcoR* I).


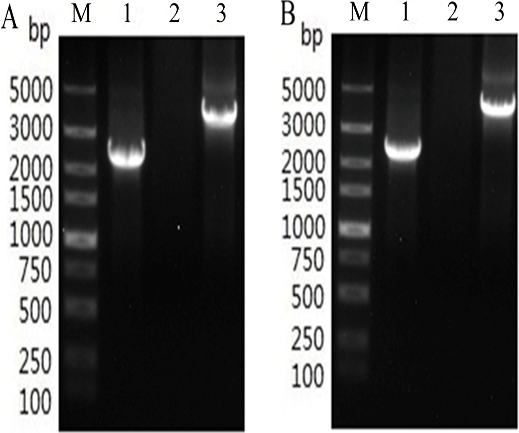


**Supplementary Figure 2. PCR identification of the bacmids.** (A) Recombinant rBacmid-E0+E2 PCR identification. M: DL5000 Marker; Lane 1: positive control; Lane 2: negative control; Lane 3: white colony. (B) Recombinant rBacmid-E2+E2 PCR identification. M: DL5000 Marker; Lane 1: positive control; Lane 2: negative control; Lane 3: white colony.
