# Supplementary figures and images for: Virus-like particles vaccines based on glycoprotein E0 and E2 of bovine viral diarrhea virus induce Humoral responses
Source: Front Microbiol. 2022 Oct 31;13:1047001. doi: 10.3389/fmicb.2022.1047001 (PMC9687372; doi:10.3389/fmicb.2022.1047001)

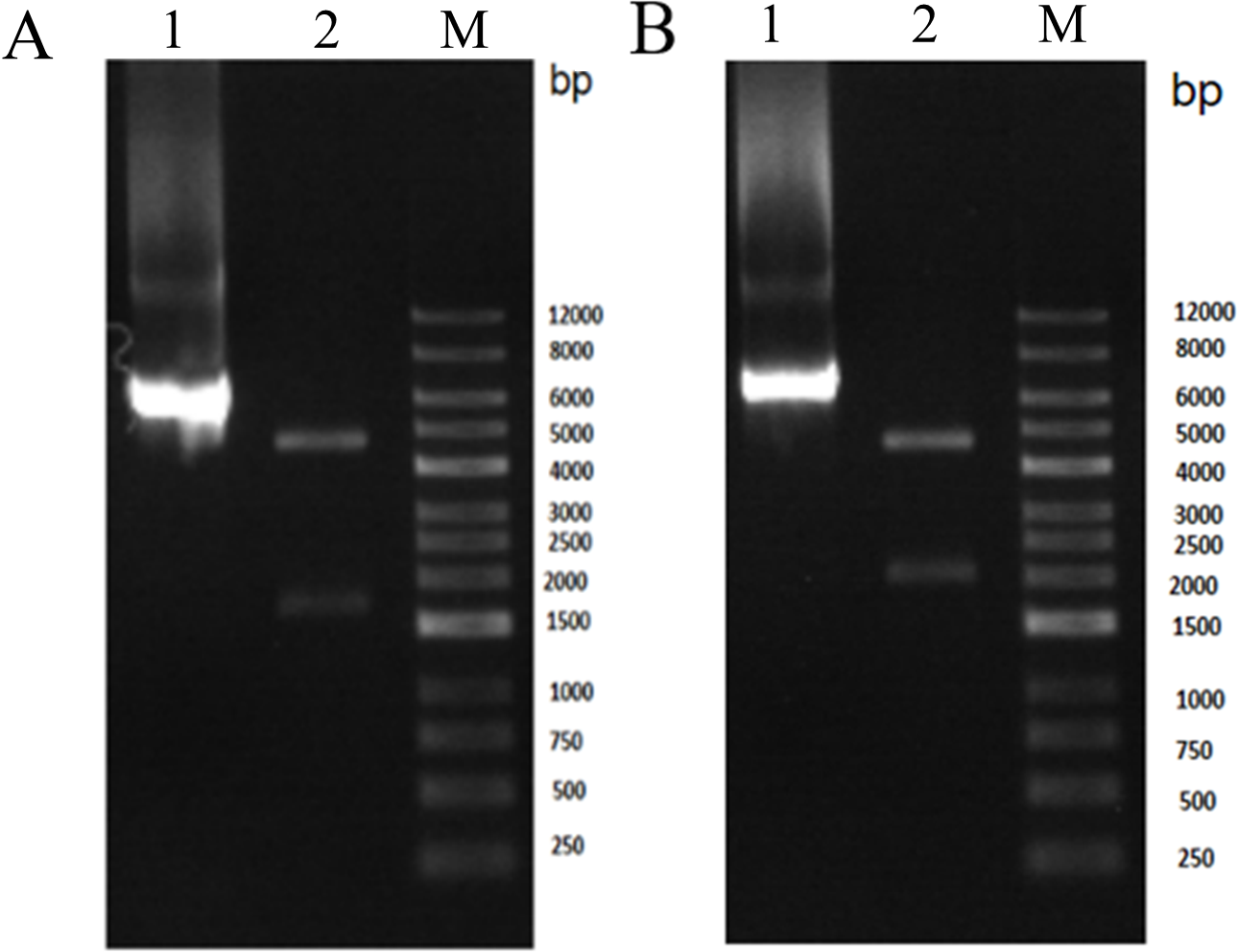

Supplement: Supplementary file 4 [file Image_1.TIF]

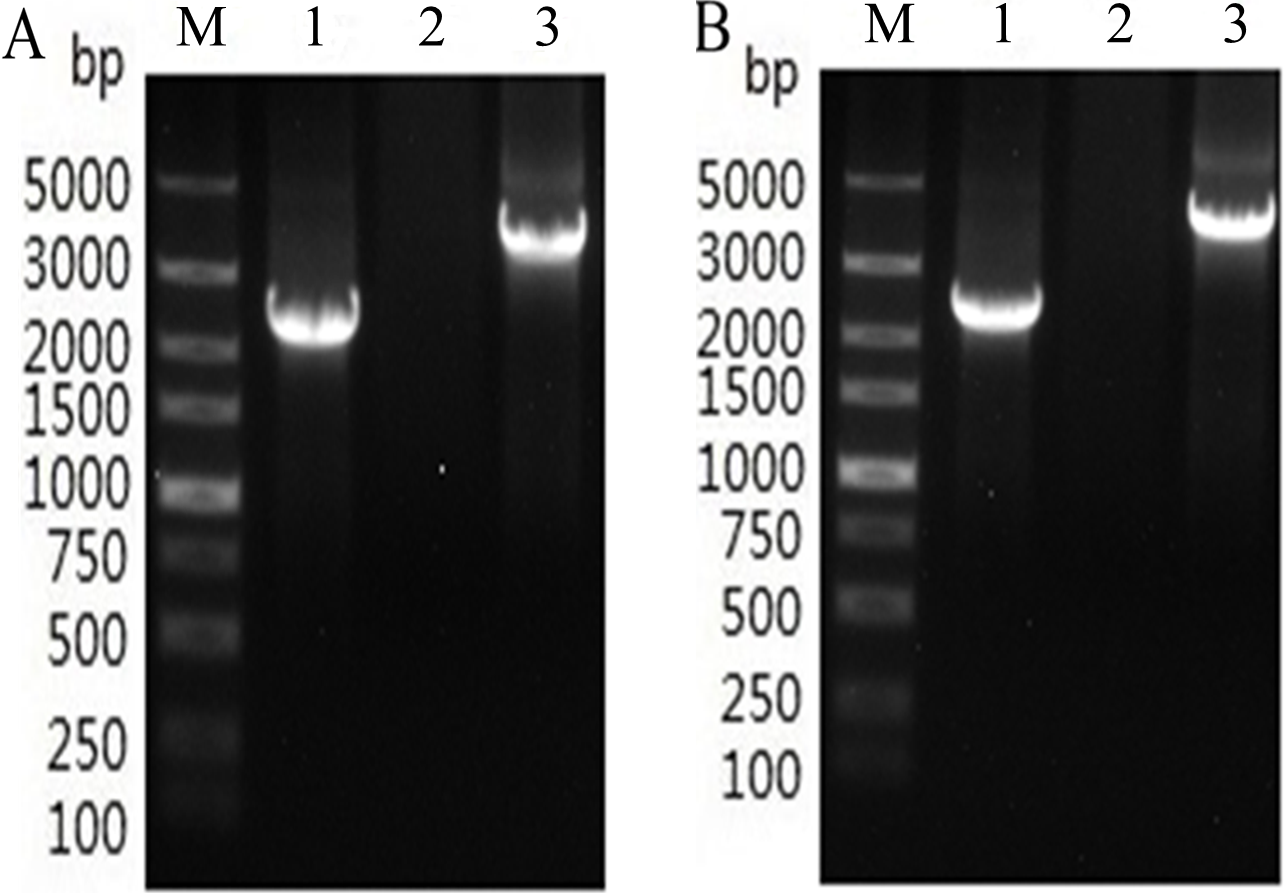

Supplement: Supplementary file 5 [file Image_2.TIF]
