# Supplementary material for: Virus-like particles vaccines based on glycoprotein E0 and E2 of bovine viral diarrhea virus induce Humoral responses
Source: Front Microbiol. 2022 Oct 31;13:1047001. doi: 10.3389/fmicb.2022.1047001 (PMC9687372; doi:10.3389/fmicb.2022.1047001)
Supplement: Supplementary file 3 [file Table_3.DOCX]

Dr. Chuangfu Chen

Shihezi University

Shihezi,832002,China

E-mail: ccf@shzu.edu.cn

October 6^th^, 2022

Dear Editor，

Firstly, we would like to thank you for your kind letter and for reviewers' constructive comments concerning our article (**Manuscript ID: "1047001"; manuscript title:** **" Virus-Like Particles Vaccines Based on Glycoprotein E0 and E2 of Bovine Viral Diarrhea Virus Induce Humoral Responses** **"**). These comments are all valuable and helpful for improving our article. All the authors have seriously discussed about all these comments. According to the reviewers' comments, we have tried best to modify our manuscript to meet with the requirements of your journal. If there are any other modifications we could make, we would like very much to modify them and we really appreciate your help.

No conflict of interest exists in submitting this manuscript, and all authors have approved the manuscript for publication. I would like to declare on behalf of my co-authors that the work described was original research that has not been published previously and is not under consideration for publication elsewhere.

Bovine viral diarrhea/mucosal disease (BVD/MD) is a viral infectious disease that seriously endangers the health of cattle herds and brings serious economic losses to the global cattle industry. Virus-like particles (VLPs) are empty shell structures without viral nucleic acid, which are similar to natural virus particles in morphology and structure. Because of their strong immunogenicity and biological activity, some of them have been used as vaccines in clinical trials. In this study, we developed a strategy to generate BVDV (E0+E2, E2+E2) VLPs using an insect baculovirus expression vector system (BEVS). The VLPs obtained were detected by immunofluorescence assay (IFA), western blotting analyses and transmission electron microscope (TEM), and the results showed that VLPs of high purity were obtained. Mice immunized with VLPs (15 μg) and Freund's adjuvant (100 μL) elicited higher BVDV- neutralizing antibody in comparison with Freund's adjuvant control (*P* < 0.0001), and even on day 21 or 35 post-prime immunization, the neutralizing antibody levels of mice immunized with E0+E2 or E2+E2 VLPs were significantly higher compared with inactivated vaccine (*P* < 0.05). A subsequent challenge reveals that the viral loads of livers, kidneys, spleens, lungs and small intestines were significantly lower compared with control (*P* < 0.0001), and the viral loads of mice immunized with E0+E2 or E2+E2 VLPs in the small intestines were significantly lower compared with inactivated vaccine (*P* < 0.05). Thus, VLPs are a promising candidate vaccine and warrants further clinical evaluation.

Thank you in advance for your consideration of our manuscript.

Thank you in advance for your consideration of our revised version.

Sincerely yours,

Chuangfu Chen, Ph.D.
